# A Pilot Study on a Possible Mechanism behind Olfactory Dysfunction in Parkinson’s Disease: The Association of TAAR1 Downregulation with Neuronal Loss and Inflammation along Olfactory Pathway

**DOI:** 10.3390/brainsci14040300

**Published:** 2024-03-22

**Authors:** Mei-Xuan Zhang, Hui Hong, Yun Shi, Wen-Yan Huang, Yi-Meng Xia, Lu-Lu Tan, Wei-Jiang Zhao, Chen-Meng Qiao, Jian Wu, Li-Ping Zhao, Shu-Bing Huang, Xue-Bing Jia, Yan-Qin Shen, Chun Cui

**Affiliations:** Department of Neurodegeneration and Injury, Wuxi School of Medicine, Jiangnan University, No. 1800, Lihu Avenue, Binhu District, Wuxi 214122, China

**Keywords:** Parkinson’s disease, olfactory impairment, olfactory pathway, TAAR1, Bcl-2/caspase3, astrocyte

## Abstract

Parkinson’s disease (PD) is characterized not only by motor symptoms but also by non-motor dysfunctions, such as olfactory impairment; the cause is not fully understood. Our study suggests that neuronal loss and inflammation in brain regions along the olfactory pathway, such as the olfactory bulb (OB) and the piriform cortex (PC), may contribute to olfactory dysfunction in PD mice, which might be related to the downregulation of the trace amine-associated receptor 1 (TAAR1) in these areas. In the striatum, although only a decrease in mRNA level, but not in protein level, of TAAR1 was detected, bioinformatic analyses substantiated its correlation with PD. Moreover, we discovered that neuronal death and inflammation in the OB and the PC in PD mice might be regulated by TAAR through the Bcl-2/caspase3 pathway. This manifested as a decrease of anti-apoptotic protein Bcl-2 and an increase of the pro-apoptotic protein cleaved caspase3, or through regulating astrocytes activity, manifested as the increase of TAAR1 in astrocytes, which might lead to the decreased clearance of glutamate and consequent neurotoxicity. In summary, we have identified a possible mechanism to elucidate the olfactory dysfunction in PD, positing neuronal damage and inflammation due to apoptosis and astrocyte activity along the olfactory pathway in conjunction with the downregulation of TAAR1.

## 1. Introduction

Parkinson’s disease (PD) has now become the second-most prevalent neurodegenerative disorder, surpassed only by Alzheimer’s disease [1]. As the global population ages, the number of individuals affected by PD is projected to exceed 17 million by the year 2040 [2]. The pathogenesis of PD is believed to stem from the substantial death of dopaminergic neurons in the substantia nigra, leading to a reduction of dopamine (DA) in the striatum. The main factors contributing to the demise of these neurons include the abnormal accumulation of α-synuclein, oxidative stress, endoplasmic reticulum stress, autophagy, apoptosis, and neuroinflammation [3]. The predominant clinical symptoms of PD patients include resting tremors and bradykinesia, among other motor dysfunctions [4]. Recent studies have indicated that, in addition to the common motor symptoms, some patients also exhibit non-motor symptoms such as constipation [5], sleep disorders [6], depression [7], and olfactory dysfunction [8,9]. Reports suggest that approximately 90% of PD patients experience olfactory dysfunction, often preceding the onset of motor symptoms [10]. Concurrently, olfactory tests in PD patients with asymmetric motor dysfunctions have also demonstrated asymmetric impairments in their sense of smell [11]. This suggests that the pathogenesis of PD may involve a progression from the peripheral to the central nervous system. The renowned Braak staging of PD also posits a pathway that begins in the nasal cavity and ultimately spreads throughout the brain [12].

Olfaction transmits along the olfactory pathway, which originates within the olfactory neurons located in the olfactory epithelium of the nasal cavity; the signals are then conveyed to the primary olfactory center, the olfactory bulb (OB) before reaching higher brain centers such as the piriform cortex (PC), the hypothalamus, and the hippocampus. Damage, such as olfactory neuron loss [13] or nasal inflammation [14], including aseptic inflammation [15], to any part of this intricate network can potentially result in olfactory dysfunction [16]. In PD patients, the reasons for olfactory dysfunction are more complex [17], encompassing the deleterious effects of abnormally accumulated α-synuclein in the olfactory cortex [18], disruptions in olfactory transmission due to imbalances in secondary neurons [19], and exposure to environmental pollutants or toxins over a longer period compared to younger, healthy individuals, which can damage the olfactory epithelium [20,21]. There are reports that PD models induced by 1-methyl-4-phenyl-1,2,3,6-tetrahydropyridine (MPTP) exhibit similar olfactory anomalies [22], which may be attributed to localized inflammation in the OB [23]; however, there is scant research on how inflammation damages the olfactory sense and the process of such damage.

Due to the olfactory dysfunction commonly found in PD, the expression of olfactory receptors along the olfactory pathway comes to mind. Trace amine-associated receptors (TAARs) are a class of G protein-coupled receptors of trace amines (TAs), the majority of which are believed to be olfactory receptors [24,25], with the exception of TAAR1 [26]. TAs are produced from amino acid precursors identical to DA, norepinephrine, and serotonin [27] and constitute a group of endogenous compounds at nanomolar concentrations that remarkably mimic the structure of classical monoamine neurotransmitters [28,29], such as β-phenylethylamine, tyramine, octopamine, and tryptamine. Alterations in the metabolism of TAs are observed in PD and other neurodegenerative diseases. PD patients exhibit a pronounced depletion of octopamine in their plasma, whereas the levels of tyramine exhibit a considerable elevation [30,31].

Although there is no evidence to suggest that TAAR1 is directly involved in the process of olfaction, our experimental results have indicated that TAAR1 is widely present along the olfactory pathway, such as the OB and the PC, which indicated the possible indirect involvement of TAAR1 in olfaction. In recent years, TAAR1 has garnered widespread attention for its regulatory effects on dopamine and dopamine receptors within the fields of schizophrenia and depression [32]. However, research in the context of neurodegenerative diseases like PD has been sparse. Indeed, TAAR1 might participate in PD pathology in some way. The mRNA of TAAR1 distributes in the substantia nigra [33], which is the key area of PD pathology. TAAR1 colocalizes with DA transporters in a subset of dopaminergic neurons within the substantia nigra [34]. Moreover, TAAR1 is able to modulate DA levels within the brain. It was found that β-phenylethylamine and tyramine could augment the release of DA from dopaminergic neurons in the substantia nigra [35], which might be due to the activation of TAAR1, resulting in the decreased DA uptake by inhibiting monoamine transporters [36].

In this study, we found that in the PD mouse model induced by MPTP, the expression of TAAR1 in olfactory-related brain regions was decreased. According to the abovementioned mechanism of TAAR1 on DA release, the reduction of TAAR1 levels in the brains of PD mice may lead to decreased DA and contribute to PD pathology. However, we obtained some new findings, which might be a clue for olfactory dysfunction in PD. It has been reported that TAAR1 is expressed in immune cells, including macrophages [37,38], astrocytes [39], and microglia [40], where it plays roles in inflammation [41]. TAAR1 has been identified as a regulator of apoptosis, exerting influence through the modulation of anti-apoptotic protein Bcl-2 levels [42]. In this study, PD mice exhibited neuroinflammation and neuronal apoptosis in several cerebral regions along the olfactory pathway, accompanied by a diminution in the expression of TAAR1. We further found that the Bcl-2/caspase 3 pathway and the activation of astrocytes contribute to neuronal apoptosis and inflammation, respectively, which might be regulated by TAAR1. Given these data, we hypothesize that TAAR1 might influence olfaction by regulating inflammation and apoptosis along the olfactory pathway in PD mice, which might contribute to the olfactory dysfunction in PD. Moreover, our study indicated the potential of TAAR1 as a novel biomarker for the early detection of PD or as a new therapeutic target for ameliorating olfactory dysfunction in PD patients.

## 2. Materials and Methods

### 2.1. Animals

Prior to the initiation of the experimental procedures, 7-week-old male C57BL/6N mice with an average weight of 20 g were acquired from the Vital River Laboratory Animal Technology Co., Ltd, Beijing, China. These animals were subsequently allotted a seven-day acclimation period within the Specific Pathogen Free (SPF) facility at the Medical Laboratory Animal Center of Jiangnan University before engaging in experimental protocols. The conditions within the facility were meticulously controlled, maintaining a temperature of 24 °C and relative humidity of approximately 55%. Furthermore, a strict 12 h light/12 h dark cycle was enforced. The mice were provided with ad libitum access to water and a standard murine chow during the acclimation and experimental phases.

After the acclimation period, the mice were randomly divided into two experimental groups: (1) Control group: intraperitoneal injection of saline only. (2) Subacute PD group: intraperitoneal injection of 1-methyl-4-phenyl-1,2,3,6-tetrahydropyridine (MPTP) for five consecutive days.

The investigation adhered to the ARRIVE (Animal Research: Reporting of In Vivo Experiments) guidelines, and the experimental protocols received approval from the Animal Ethics Committee at Jiangnan University. (JN. No20190330c681231[37]).

### 2.2. MPTP Treatment

Following the procedural steps documented in the references [43], we constructed subacute PD mice models via intraperitoneal injections of MPTP-HCl (M0896, Sigma-Aldrich, St. Louis, MO, USA). For the subacute PD models, C57BL/6N mice were administered intraperitoneal injections of MPTP at a dosage of 30 mg/kg in a volume of 10 mL/kg of body weight once daily for a duration of five days. The control group mice were injected with an equivalent volume of saline solution at corresponding time intervals.

### 2.3. Sample Collection

To procure fresh brain tissues, mice underwent deep anesthesia induced by isoflurane, followed by transcardial perfusion with 20 mL of ice-cold, sterile saline per animal. The OB, cortex, hypothalamus, and hippocampus were rapidly excised and immediately preserved at −80 °C. For immunofluorescence or immunohistochemistry analysis, the mice were perfused transcardially with phosphate-buffered saline (PBS), succeeded by 4% paraformaldehyde (PFA) in 0.01 M phosphate buffer at pH 7.4. Subsequently, the whole brain was fixed in 4% PFA at 4 °C overnight, then incubated in 20% and 30% sucrose sequentially each for 24 h at 4 °C, and ultimately embedded in Optimal Cutting Temperature (O.C.T.) compound (Tissue-Tek, Torrance, CA, USA). Coronal sections of 10 μm in thickness were prepared using a CM1950 cryostat microtome (Leica, Wetzlar, Germany).

### 2.4. Immunofluorescence Staining

Frozen coronal sections of the olfactory bulb and midbrain underwent a process of antigen retrieval by being submerged in a 0.01 M sodium citrate solution with a pH level of 6.0, followed by a thrice rinse in PBS solution. Subsequently, these slices were treated with a PBS mixture enhanced by 0.3% *v*/*v* Triton X-100 and a 10% *v*/*v* solution of goat serum, maintained at 37 °C for 30 min. The primary antibodies utilized included rabbit anti-NeuN (1:500, ABN78, Sigma-Aldrich, St. Louis, MO, USA), rabbit anti-CD68 (1:1000, BA3638, Boster, Wuhan, China), rabbit anti-GFAP (1:1000, GB11096-100, Servicebio, Beijing, China), mouse anti-Iba-1 (1:1000, GB12105-100, Servicebio, Beijing, China), and mouse anti-TAAR1 (1:200, sc-514311, Santa Cruz, Paso Robles, CA, USA), all of which were applied to the brain sections and left to incubate at a cool temperature of 4 °C throughout the night. The subsequent identification of these primary antibodies was facilitated through the application of suitable secondary antibodies; these were either coupled with FITC-conjugated goat anti-mouse IgG (1:1000, A0568, Beyotime, Shanghai, China) or CY3-conjugated goat anti-rabbit IgG (1:1000, A0516, Beyotime, Shanghai, China). DAPI was employed to counterstain the nuclei. Fluorescence images were captured using a Zeiss Axio Imager Z2 microscope (Thuringia, Germany), and quantitative analysis was performed utilizing ImageJ software (2.14.0/1.54f).

### 2.5. Western Blot Analysis

Total protein was isolated from 10 mg of the fresh tissue homogenized in 100 μL of RIPA lysis buffer (P0013B, Beyotime, Shanghai, China) containing 1% phenylmethanesulfonyl fluoride (PMSF) (ST506, Beyotime, Shanghai, China) and 2% phosphatase inhibitor (P1081, Beyotime, Shanghai, China). The homogenate was centrifuged at 13,000 rpm and 4 °C for 5 min. The protein concentration in the collected supernatant was quantified using a BCA Protein Quantification Kit (E112-01, Vazyme, Nanjing, China). The proteins were then denatured by heating in SDS-PAGE loading buffer, 4× (with DTT) (P1015, Solarbio, Beijing, China) at 100 °C for 15 min. A total of 30 μg of the total protein was separated on a 10% and 12% SDS-PAGE gel, transferred to PVDF membranes (ISEQ00010, Millipore, Billerica, MA, USA), and subsequently blocked with 5% skim milk (5% bovine serum albumin for phosphorylated protein) at room temperature for 2 h before being incubated with specific antibodies overnight at 4 °C. The following primary antibodies were used to probe the proteins: rabbit anti-CD68 (1:1000, BA3638, Boster, Wuhan, China), rabbit anti-TNF-α (1:1000, 11948S, CST, Danvers, MA, USA), rabbit anti-p-NF-κB (1:1000, 3033S, CST, Danvers, MA, USA), mouse anti-TAAR1 (1:500, sc-514311, Santa Cruz, Paso Robles, CA, USA), mouse anti-Bcl-2 (1:1000, 610538, BD, Franklin Lakes, NJ, USA), rabbit anti-caspase3 (1:1000, 9662s, CST, Danvers, MA, USA), rabbit anti-β-tubulin (1:2000, 10068-1-AP, Proteintech, Wuhan, China), and rabbit anti-GAPDH (1:8000, 10494-1-AP, Proteintech, Wuhan, China). Goat anti-rabbit IgG (1:8000, BA1054, Boster, Wuhan, China) and goat anti-mouse IgG (1:8000, BA1051, Boster, Wuhan, China) conjugated with horseradish peroxidase were used as secondary antibodies. Protein bands were detected following incubation with an Enhanced Chemiluminescence Substrate Kit (E411-04, Vazyme, Nanjing, China) for 1 min and were visualized using a Tanon 5200 Gel Image System (Shanghai, China). Densitometric analysis was conducted by ImageJ software (2.14.0/1.54f).

### 2.6. Quantitative Real-Time PCR (qRT-PCR) Analysis

Total RNA was extracted from the fresh tissues by using a TRIzol™ reagent (15596018, Invitrogen, Carlsbad, CA, USA), following the protocol provided by the manufacturer. cDNA synthesis was conducted from the extracted RNA using the PrimeScript™ RT reagent kit (RR036A, Takara, Kusatsu, Shiga, Japan). Quantitative real-time PCR (qRT-PCR) assays were executed on a LightCycler 480 II instrument (Roche, Boston, MA, USA) utilizing SYBR^®^ Premix Ex Taq™ II (RR820A, Takara, Kusatsu, Shiga, Japan) with specifically validated primer pairs sourced from PrimerBank (except *Taar1*, which was sourced from a reference [44]). Expression levels of target genes were normalized to *Gapdh* as an internal control, and the relative quantification of mRNA levels was determined by employing the 2^−ΔΔCt^ method. The primer sequences used in this study are listed in Table 1.

### 2.7. Data Acquisition and Analysis

To ascertain whether human-derived TAAR1 undergoes alterations in PD, we retrieved pertinent data from the Gene Expression Omnibus (GEO) database that employed human cell samples. The data was retrieved for 16 samples from the GSE198009 within the GEO database [45], and the expression levels of *TAAR1* were quantified in both the control group and the model group. Subsequently, SPSS 26.0 software was employed to conduct a differential analysis of the results.

### 2.8. Immunohistochemical Staining

Immunohistochemical analysis was employed to ascertain the expression level of TAAR1 within the murine brains. Frozen coronal sections of the olfactory bulb and midbrain were immersed in 0.01 M sodium citrate buffer (pH 6.0) for antigen retrieval at 95 °C for 45 min and washed thrice in PBS. These slices were treated with a PBS mixture enhanced by 0.3% *v*/*v* Triton X-100 and a 10% *v*/*v* solution of goat serum, maintained at 37 °C for 30 min, then incubated with the primary antibody mouse anti-TAAR1 (1:200, sc-514311, Santa Cruz, Paso Robles, CA, USA) at 4 °C overnight. Subsequently, the sections were washed thrice in PBS and were further incubated with HRP-labeled poly peroxidase-anti-mouse IgG by using the Universal Two-Step Detection Kit (PV-9000, ZSGB-Bio, Beijing, China); the color was developed by the AEC Enzyme Substrate Kit (ZLI-9036, ZSGB-Bio, Beijing, China), according to the manufacturer’s instructions. The procedure concluded with counterstaining using hematoxylin (AR0005, Boster, Wuhan, China) and was routinely mounted. Images were captured with a Nikon Eclipse 80i microscope (Japan) and quantified with ImageJ software (2.14.0/1.54f).

### 2.9. Statistical Analysis

Data are expressed as mean ± standard error of the mean (SEM). Statistical comparisons between groups were conducted using an independent-sample *t*-test. A *p*-value of less than 0.05 was deemed to denote statistical significance. All statistical evaluations were performed by GraphPad Prism software, version 8.0.

## 3. Results

### 3.1. Neuronal Loss and Neuroinflammation Were Increased in the OB and PC in PD Mice

**Figure 1:**
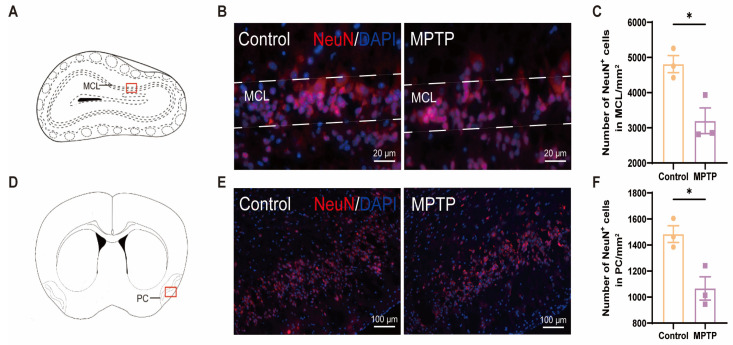
The neuronal population declined in the olfactory bulb (OB) and the piriform cortex (PC) of the PD mice. (**A**) Schematic of the mouse OB coronal section, with the red box indicating the area depicted in (**B**). (**B**) Immunofluorescence staining of NeuN in the mitral cell layer (MCL) of the OB. Scale bar = 20 μm. (**C**) The neuron counts within the MCL of the OB were recorded as cells/mm^2^, *n* = 3. (**D**) Schematic of the mouse midbrain coronal section, with the red box highlighting the area shown in (**E**). (**E**) Immunofluorescence staining of NeuN at the PC region. Scale bar = 100 μm. (**F**) The neuron counts within the PC were recorded as cells/mm^2^, *n* = 3. Statistical comparison by independent-sample *t*-test. Data are expressed as means ± SEM. * *p* < 0.05.

The olfactory impairments observed in PD patients may be attributed to a reduction in the number of neurons within the brain regions associated with olfaction [46]. According to the immunofluorescence staining of NeuN (a marker for neurons), a significant decrease in the number of neurons within both the OB and the PC was observed in the MPTP-induced PD group (Figure 1).

**Figure 2:**
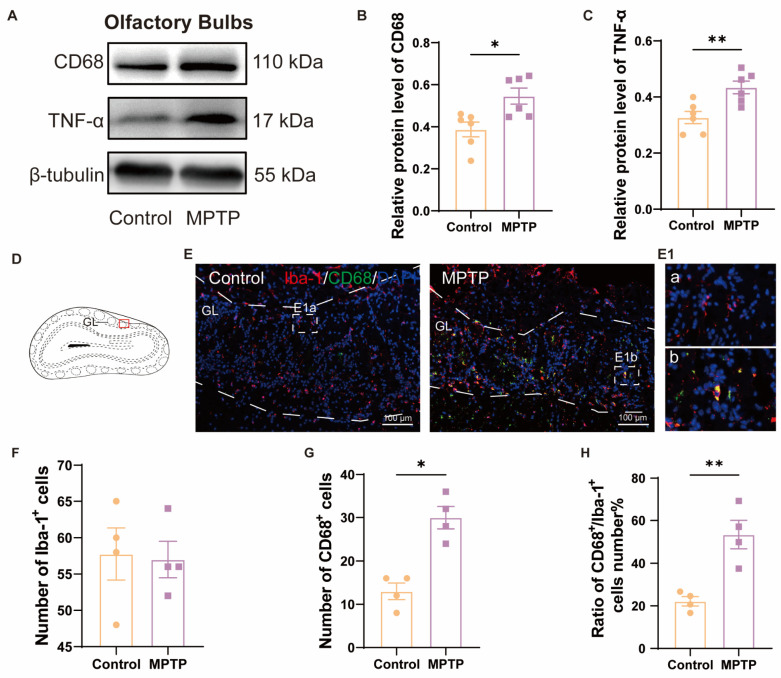
The level of neuroinflammation within the OB of PD mice was elevated. (**A**) Representative western blot images for CD68 and TNF-α in the OB. (**B**) Quantitative assessment of CD68 levels was normalized to β-tubulin expression, *n* = 6. (**C**) Quantitative assessment of TNF-α levels was normalized to β-tubulin expression, *n* = 6. (**D**) Schematic of the mouse OB coronal section, with the red box indicating the area shown in (**E**). (**E**) Immunofluorescent co-staining of Iba-1 and CD68 in the glomerular layer (GL) of the OB. Scale bar = 100 μm; E1a and E1b are magnified images within the dashed box for the control and the PD group, respectively. (**F**) Quantification of Iba-1^+^ cells in the GL of the OB. (**G**) Quantification of CD68^+^ cells in the GL of the OB. (**H**) The ratio of CD68^+^ to Iba-1^+^ cells number in the GL of the OB, *n* = 4. Statistical comparison by independent-sample *t*-test. Data are expressed as means ± SEM. * *p* < 0.05, ** *p* < 0.01.

We further detected neuroinflammation in the aforementioned regions, attributed to its role in inducing neuronal death. In the OB, western blot analyses demonstrated heightened levels of CD68 and TNF-α in the PD group (Figure 2A–C). Immunofluorescence results corroborated this finding. Although the number of Iba-1^+^ cells (a marker for microglia) in the OB did not show a difference between the two groups (Figure 2D–F), the number of CD68^+^ cells (a marker for activated microglia) and the ratio of CD68^+^ cells to Iba-1^+^ cells were increased in the PD group (Figure 2D,E,G,H). These indicated that there was inflammation in the OB of the PD group. Morphological comparisons of the microglia revealed that the control group possessed more branched, resting-state microglia (Figure 2E(1a)), whereas the PD group possessed fewer branched and activated microglia with an amoeboid appearance (Figure 2E(1b)).

**Figure 3:**
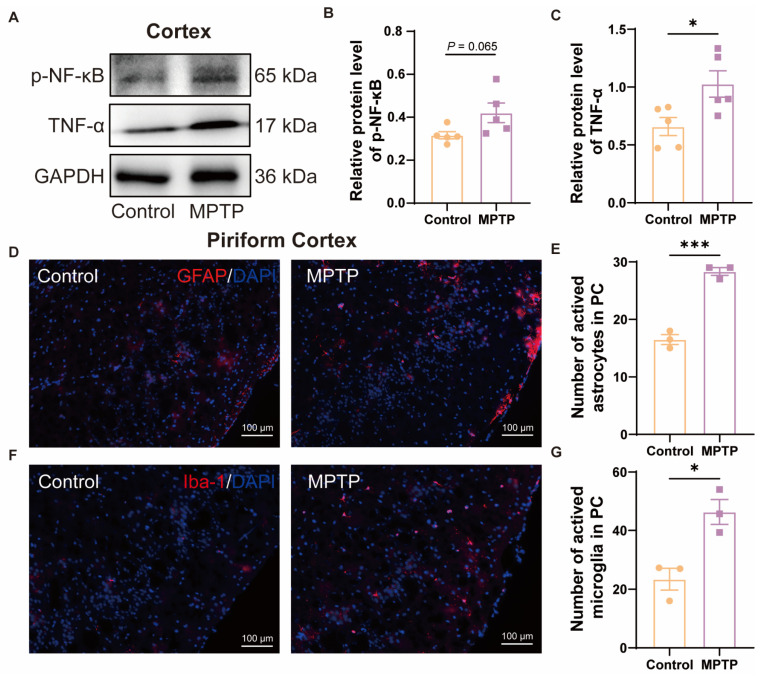
The level of neuroinflammation within the cortex of subacute PD mice was increased. (**A**) Representative western blot images for p-NF-κB and TNF-α in cortical tissue. (**B**) Quantitative assessment of p-NF-κB levels was normalized to GAPDH expression, *n* = 5. (**C**) Quantitative assessment of TNF-α levels was normalized to GAPDH expression, *n* = 5. (**D**) Immunofluorescent staining of GFAP at the PC region. Scale bar = 100 μm. (**E**) Quantification of GFAP^+^ cells in the PC, *n* = 3. (**F**) Immunofluorescent staining of Iba-1 in the PC region. Scale bar = 100 μm. (**G**) Quantification of Iba-1^+^ cells in the PC, *n* = 3. Statistical comparison by independent-sample *t*-test. Data are expressed as means ± SEM. * *p* < 0.05, *** *p* < 0.001.

In the cortex, western blot results demonstrated that the PD group exhibited higher expression of p-NF-κB and TNF-α (Figure 3A–C). According to the immunofluorescence analysis, an increased number of GFAP^+^ cells (a marker for active astrocytes) and Iba-1^+^ cells in the PC of the PD group was observed (Figure 3D–G). These also indicated more pronounced neuroinflammation in the PC of the PD group than in the control group.

All these findings suggest that the olfactory dysfunction observed in PD mice may be associated with neuronal damage within the OB and PC, coupled with elevated levels of neuroinflammation.

### 3.2. Taar1 mRNA Was Down-Regulated from the OB to the Hypothalamus along the Olfactory Pathway in PD Mice

**Figure 4:**
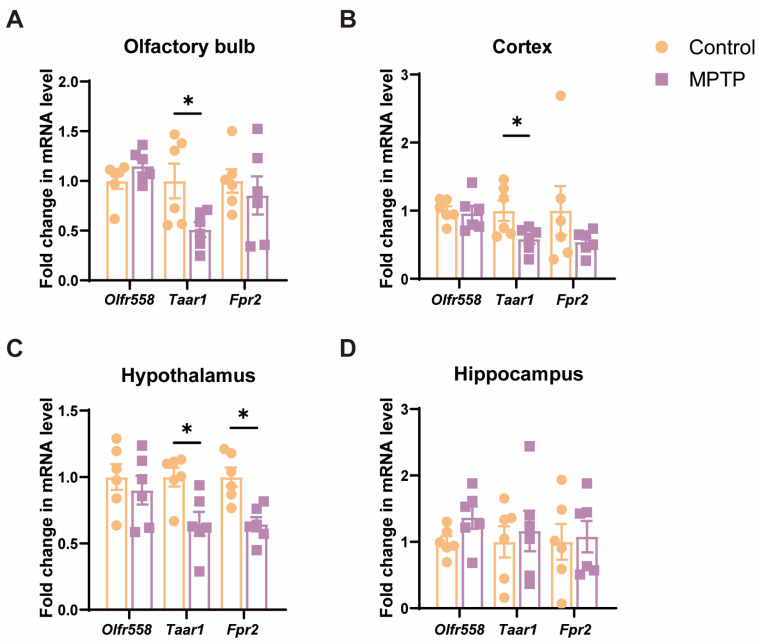
The mRNA levels of three olfactory- or inflammation-related molecules in various brain regions along the olfactory pathway. (**A**) The OB. (**B**) The cortex. (**C**) The hypothalamus. (**D**) The hippocampus. Statistical comparison by independent-sample *t*-test. Data are expressed as means ± SEM, *n* = 6. * *p* < 0.05.

Since cell death and inflammation along the olfactory pathway were detected, we examined the mRNA levels of several molecules, including the olfactory receptor 558 (*Olfr558*), *Taar1* [40,42], and formyl peptide receptor 2 (*Fpr2*) [47], which are either involved in the above-mentioned cellular events or olfaction. The results indicated that, among these molecules, the levels of *Taar1* were significantly diminished in several olfaction-related regions, including the OB, the cortex, and the hypothalamus in PD mice (Figure 4A–C). *Olfr558* displayed no significant changes in these regions between the two groups (Figure 4A–C). *Fpr2* was only notably decreased in the hypothalamus in PD mice. Additionally, these molecules did not show a significant difference in the hippocampus, which is a region far away from the origin of the olfactory pathway (Figure 4D). Therefore, *Taar1* seems to be involved in olfaction in PD mice.

**Figure 5:**
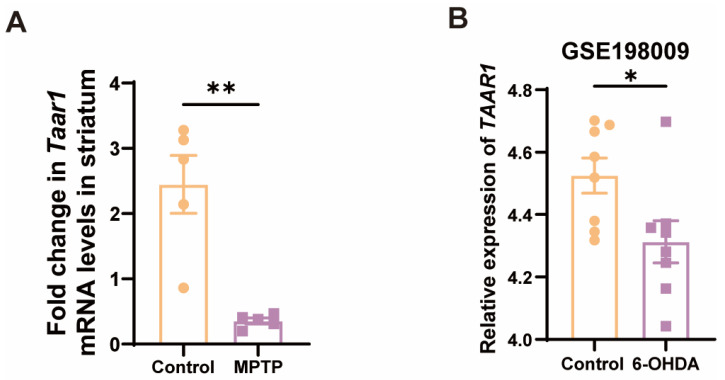
*Taar1* mRNA was decreased in the striatum of PD mice, and its decline was highly correlated with PD. (**A**) The mRNA levels of *Taar1* in the striatum of the MPTP-induced subacute PD mice, *n* = 5. (**B**) The transcriptomic levels of *TAAR1* decreased in an in vitro PD model induced by 6-OHDA treatment compared to the control. (GSE198009 [45], *n* = 8). Statistical comparison by independent-sample *t*-test. Data are expressed as means ± SEM. * *p* < 0.05, ** *p* < 0.01.

Given that the nigrostriatal system of the midbrain is the principal locus in PD, we further examined the *Taar1* mRNA levels within the striatum of the subacute PD mice. As anticipated, there was a discernible downregulation of *Taar1* in the striatum of PD mice (Figure 5A). To ascertain whether human-derived TAAR1 undergoes alterations in PD, we consulted the GEO database, specifically the GSE198009 dataset, which contrasts the transcriptome data differences between an in vitro PD model, which was constructed by stimulating SH-SY5Y cells with 6-hydroxydopamine (6-OHDA), and a control group [45]. This dataset revealed a significant decrease in the expression of *TAAR1* in the in vitro PD model (Figure 5B). This result indicated that the expression of human-derived TAAR1 also varies within the PD model, thereby negating the possibility that such changes are exclusive to murine species. These findings suggest a high correlation between the downregulation of *TAAR1* expression and PD.

### 3.3. TAAR1 Protein Was Down-Regulated from the OB to Hypothalamus along the Olfactory Pathway in PD Mice

**Figure 6:**
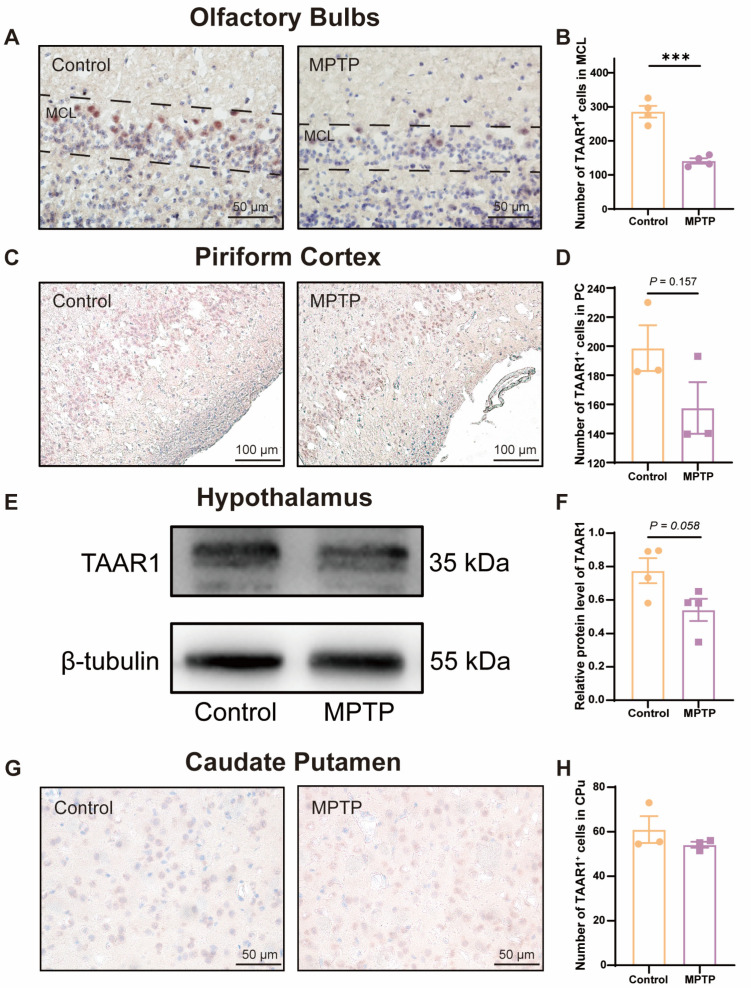
TAAR1 protein levels declined along the olfactory pathway in subacute PD mice. (**A**) Immunohistochemical staining of TAAR1 at the MCL of the OB. Scale bar = 50 μm. (**B**) Quantification of TAAR1^+^ cells within the MCL of the OB, *n* = 4. (**C**) Immunohistochemical staining of TAAR1 in the PC region. Scale bar = 100 μm. (**D**) Quantification of TAAR1^+^ cells within the PC, *n* = 3. (**E**) Representative western blot image for TAAR1 in the hypothalamus. (**F**) Quantitative assessment of TAAR1 levels was normalized to β-tubulin expression, *n* = 4. (**G**) Immunohistochemical staining of TAAR1 at the CPu of the striatum. Scale bar = 50 μm. (**H**) Quantification of TAAR1^+^ cells within the CPu, *n* = 3. Statistical comparison by independent-sample *t*-test. Data are expressed as means ± SEM. *** *p* < 0.001.

To further substantiate whether the levels of the TAAR1 protein along the olfactory pathway were reduced in the subacute PD mice, we employed immunohistochemical staining and western blot to assess TAAR1 protein levels within the OB (Figure 6A,B), the PC (Figure 6C,D), the hypothalamus (Figure 6E,F), and the caudate putamen (CPu) (Figure 6G,H). We observed an abundance of TAAR1^+^ cells distributed throughout the OB, and TAAR1^+^ cells decreased in PD mice (Figure 6A,B), which was consistent with the qRT-PCR findings within the OB.

TAAR1 protein levels within the PC (Figure 6C,D) and the hypothalamus (Figure 6E,F) did not show a significant difference between the two groups but showed a decreased trend in PD mice (*p* = 0.157 for the PC, and *p* = 0.058 for the hypothalamus). In the CPu of the striatum, TAAR1^+^ cells did not show a difference in number between the two groups (Figure 6G,H). These findings indicate that the decline in TAAR1 protein levels occurred exclusively within olfaction-related areas and progressed along the olfactory pathway from the nasal region to the brain in PD mice.

### 3.4. The Neuronal Death in the OB and the PC in PD Mice Might Be Related to the Enhancement of Apoptosis Modulated by the Bcl-2/caspase3 Pathway

**Figure 7:**
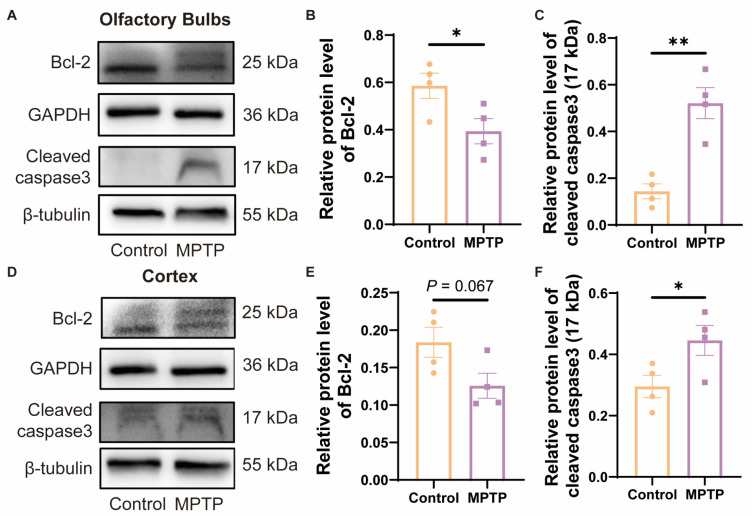
The expression of Bcl-2/caspase3 within the OB and the cortex of subacute PD mice. (**A**) Representative Western blot image for Bcl-2 and cleaved caspase3 in the OB. (**B**) Quantitative assessment of Bcl-2 levels in the OB was normalized to GAPDH expression, *n* = 4. (**C**) Quantitative assessment of cleaved caspase3 (17 kDa) levels in the OB was normalized to β-tubulin expression, *n* = 4. (**D**) Representative western blot image for Bcl-2 and cleaved caspase3 in the cortex. (**E**) Quantitative assessment of Bcl-2 levels in cortex was normalized to GAPDH expression, *n* = 4. (**F**) Quantitative assessment of cleaved caspase3 (17 kDa) levels in cortex was normalized to β-tubulin expression, *n* = 4. Statistical comparison by independent-sample *t*-test. Data are expressed as means ± SEM. * *p* < 0.05, ** *p* < 0.01.

Studies have indicated that TAAR1 modulates the levels of cellular apoptosis by regulating the expression of the anti-apoptotic protein Bcl-2 [42]. To investigate whether the reduction in neuronal numbers in the OB and the PC was due to apoptosis regulated by Bcl-2, which might be a consequence of the decrease in TAAR1 in PD mice, we examined the protein levels of Bcl-2 by western blot. Since Bcl-2 can inhibit the cleavage activation of caspase3, as the downstream protein of Bcl-2, cleaved caspase3 in these regions was also detected. In the OB, Bcl-2 decreased, and cleaved caspase3 increased in PD mice (Figure 7A–C). In the cortex, Bcl-2 did not show a difference between the two groups, while PD mice showed a decrease trend (Figure 7D,E) (*p* = 0.067), and cleaved caspase3 increased in PD mice (Figure 7D,F). According to these findings, the decrease of Bcl-2 induced higher levels of cleaved caspase3 in PD mice, which is consistent with previous reports [42] (Figure 7A,D). Therefore, the Bcl-2/caspase3 pathway might contribute to the reduced neurons in the OB and PC in PD mice, which might be regulated by TAAR1.

### 3.5. TAAR1 Levels Increased in Astrocytes within the OB and the PC in PD Mice

**Figure 8:**
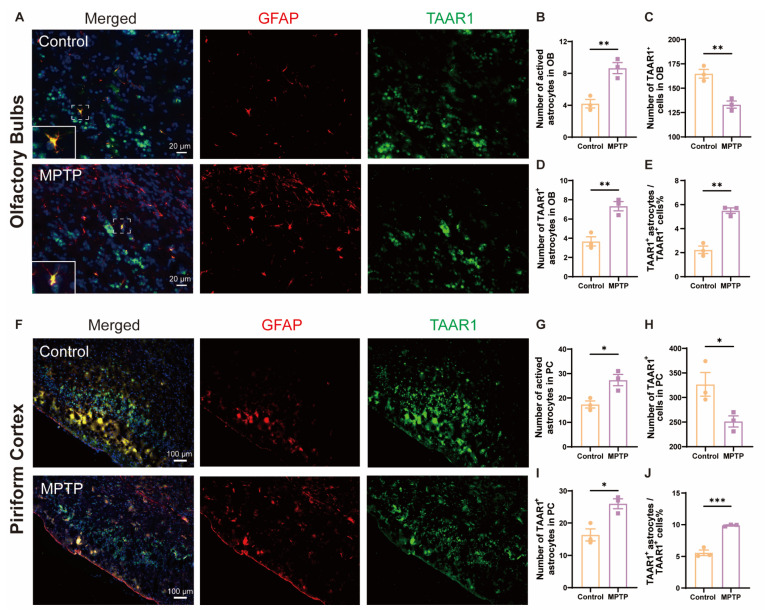
TAAR1 expression was increased in astrocytes within the OB and the PC in PD mice. (**A**) Immunofluorescent co-staining of GFAP (red) and TAAR1 (green) in the OB. Within the dashed box, TAAR1^+^/GFAP^+^ double-positive cells appear in yellow. Scale bar = 20 μm. (**B**) Quantification of GFAP^+^ cells in the OB, *n* = 3. (**C**) Quantification of TAAR1^+^ cells in the OB, *n* = 3. (**D**) Quantification of TAAR1^+^ astrocytes in the OB, *n* = 3. (**E**) The ratio of TAAR1^+^ astrocytes to TAAR1^+^ cells in the OB, *n* = 3. (**F**) Immunofluorescent co-staining of GFAP (red) and TAAR1 (green) in the PC. Scale bar = 100 μm. (**G**) Quantification of GFAP^+^ cells in the PC, *n* = 3. (**H**) Quantification of TAAR1^+^ cells in the PC, *n* = 3. (**I**) Quantification of TAAR1^+^ astrocytes in the PC, *n* = 3. (**J**) The ratio of TAAR1^+^ astrocytes to TAAR1^+^ cells in the PC, *n* = 3. Statistical comparison by independent-sample *t*-test. Data are expressed as means ± SEM. * *p* < 0.05, ** *p* < 0.01, *** *p* < 0.001.

Inflammation is a main contributor to cell death; TAAR1 participates in the regulation of inflammation. To explore whether the neuroinflammation observed in the OB and the PC of PD mice was associated with alterations in TAAR1, we employed the immunofluorescence co-staining of GFAP and TAAR1 in the OB and the PC. In the OB, consistent with the above results, the number of GFAP^+^ cells increased, and the TAAR1^+^ cells decreased in PD mice (Figure 8A–C). Not only did the number of TAAR1^+^/GFAP^+^ double positive cells increase (Figure 8D), but the ratio of TAAR1^+^/GFAP^+^ cells to TAAR1^+^ cells also increased (Figure 8E). Similar to the OB, in the PC, the number of GFAP^+^ cells increased, and the TAAR1^+^ cells decreased in PD mice (Figure 8F–H). Not only did the number of TAAR1^+^/GFAP^+^ double positive cells increase (Figure 8I), but the ratio of TAAR1^+^/GFAP^+^ cells to TAAR1^+^ cells also increased (Figure 8J). These results indicated that TAAR1 levels were increased in the astrocytes within the OB and the PC in PD mice, which might modulate the enhancement of neuroinflammation in these regions [48]. 

## 4. Discussion

Olfactory dysfunction is ubiquitous in PD patients and serves as a potential clinical marker for the early diagnosis of PD [49,50]. However, the underlying mechanisms remain elusive [10]. In late-stage α-synuclein transgenic mice, the accumulation of α-synuclein in the OB leads to neuronal damage, which might account for the olfactory dysfunction in PD [51]. Nonetheless, this hypothesis could not elucidate the olfactory deficits commonly found in early-stage PD patients. Herein, we propose an alternative mechanism for olfactory dysfunction in PD, suggesting that neuronal apoptosis and neuroinflammation in the brain regions along the olfactory pathway, such as the OB and the PC, are responsible for the olfactory impairments seen in PD patients. This phenomenon may also be associated with a reduction in TAAR1 in these regions.

Much like vision and hearing, the faculty of smell invariably declines with age [52], attributable to factors such as decreased regenerative capacity [53], cellular apoptosis [54], and disruptions in neural transmission [55]. Different from normal age-related olfactory decline, the above process is further exacerbated in PD patients due to neurodegenerative or other PD-related pathological lesions [16]. For example, the dopaminergic neuronal damage by 6-OHDA injection in the OB resulted in olfactory dysfunction [46]. In individuals with PD, a specific correlation exists between the thinning of the cortical layers in PC and the impairment of olfactory capabilities [56]. Inflammation is a common reason for olfactory dysfunction [57,58], while severe inflammation is always found in PD patients [59]; the inflammation environment further inflicts damage on the structure and function of the olfactory epithelium and the OB [60]. In our study, apoptosis and inflammation in the OB and the PC of MPTP-induced subacute PD mice were also found, manifested as the activation of microglia or astrocytes in the OB and PC. MPTP-induced activation of NLRP3 might contribute to this [23]. Since there are limited studies about olfactory dysfunction in PD, the cause actually remains elusive. The olfactory ability was not examined in our study, which makes the relationship between olfactory dysfunction and PD obscure. However, it has been reported that both acute and subacute PD mice induced by MPTP exhibited olfactory dysfunction [22,23,61], which helps to support the relationship between olfactory dysfunction and PD.

To explore the underlying mechanism of apoptosis and inflammation along the olfactory pathway in MPTP-induced subacute PD mice, several molecules that are involved in these processes and expressed along the olfactory pathway were examined. TAAR1 emerged as the only one whose mRNA was down-regulated in every brain region along the olfactory pathway and striatum. However, among these regions, only the protein levels of TAAR1 in the OB exhibited a significant change, while in the PC and hypothalamus, there was merely a downward trend (*p* = 0.157, *p* = 0.058, respectively). According to the biological synthesis process, the mRNA level of *TAAR1* might be the first affected, followed by the protein level. Furthermore, the PC and hypothalamus are situated deeper along the olfactory pathway compared to the OB, which suggests that the reduction in TAAR1 protein expression might be a progressive process, which might begin in the OB and gradually spread to deeper brain areas over time. Moreover, consistent with our findings, the downregulation of TAAR1 in another PD model was also reported. In Park2 gene knockout rats, there is a reduction in the levels of TAAR1 in the striatum [62]. Despite the protein level of TAAR1 in the striatum not being changed in our MPTP-induced subacute PD mice compared to control, it might be due to the different influences on TAAR1 expression according to the different protocols to induce PD models.

The decrease of TAAR1 in olfactory-related brain regions may lead to olfactory dysfunction in MPTP-induced PD mice through several mechanisms: (1) Regulating cellular apoptosis: In spinal cord-injured rats, when TAAR1 was activated, the apoptosis in spinal motor neurons was inhibited, and the recovery of hindlimb motor functions was facilitated [63]. TAAR1 can enhance the expression of Bcl-2 via the cAMP-ERK1/2-dependent pathway and abrogate the activation of the caspase3 apoptotic cascade, thereby exerting neuroprotective effects [42]. Consequent to these findings, the reduction of TAAR1 along the olfactory pathway in our study might result in Bcl-2/caspase-3 alterations and lead to neuronal damage. (2) Regulating the activity of astrocytes: Activation of TAAR1 in astrocytes significantly downregulates the level of the glutamate transporter EAAT-2, leading to an accumulation of glutamate and subsequent cytotoxicity [39,64] due to the corresponding influx of Ca^2+^ into the cells to trigger apoptotic signals and the release of IL-1β, resulting in apoptosis and neuroinflammation [48]. The following two possible mechanisms are speculated according to previous reports and require further investigation: (3) Regulating the activity of microglia: Activation of TAAR1 in microglia can suppress the release of proinflammatory factors such as IL-6, TNF-α, NF-κB, MCP1, and MIP1, while simultaneously promoting the release of anti-inflammatory mediators like IL-10, thus fulfilling an anti-inflammatory role [40]. (4) Regulating dopaminergic neurons: As previously reported, dopaminergic neuronal damage in the OB could result in olfactory dysfunction [46], which indicates that dopaminergic neurons might also participate in olfaction.

To elucidate the role of TAAR1 in olfactory dysfunction in PD, some limitations in our study should be solved by further studies since TAAR1 might play multiple roles in different cells. It has been reported that the functions of TAAR1 in glial cells and neurons may differ or even be diametrically opposed [41,65,66]; we did not detect changes in TAAR1 within microglia. Due to these reasons, the link between TAAR1 and PD pathology is still not clear in the present study. TAAR1 knockout animals might give some clues, but most of the previous studies were conducted in TAAR1 conventional knockout mice [65,67,68]. A cell-type-specific or region-specific TAAR1 knockout mice should be used in future studies.

## 5. Conclusions

In this study, we have discerned a reduction in TAAR1 expression within several brain regions along the olfactory pathway in MPTP-induced subacute PD mice, which might result in the activation of apoptosis and neuroinflammation by regulating the Bcl-2/caspase3 pathway and astrocytes ability respectively. These alterations may contribute to neuronal death in olfaction-related brain regions and olfactory dysfunction in PD. Thus, TAAR1 seems to be an interesting molecule involved in PD olfactory dysfunction. Additional profound studies are needed to validate its value as a biomarker or therapeutic target in PD. Early TAAR1 agonist treatment within the nose might alleviate neuronal damage and olfactory dysfunction along the olfactory pathway in PD.

## Figures and Tables

**Table 1:** qRT-PCR primers used in this study.

Gene Name (PrimerBank ID)	Forward and Reverse Sequence
*Gapdh* (6679937a1)	Forward: 5′-AGGTCGGTGTGAACGGATTTG-3′
	Reverse: 5′-TGTAGACCATGTAGTTGAGGTCA-3′
*Olfr558* (22128779a1)	Forward: 5′-TCAATAGCAATGAATCCAGTGCC-3′
	Reverse: 5′-GCACAGCAATAAGGTAGAGGGAA-3′
*Taar1* [44]	Forward: 5′-AGGACAAGCAAGGTCAATCAATCG-3′
	Reverse: 5′-AGAACGGGCACCAGCATACG-3′
*Fpr2* (6679853a1)	Forward: 5′-GAGCCTGGCTAGGAAGGTG-3′
	Reverse: 5′-TGCTGAAACCAATAAGGAACCTG-3′

## Data Availability

The data that support the findings of this study are available from the corresponding author. The data are not publicly available due to ethical and privacy constraints.
